# Microbiome Notes—Building a Library of Descriptive Microbial Ecology

**DOI:** 10.1111/1758-2229.70085

**Published:** 2025-03-26

**Authors:** Gareth Jenkins, Eric S. Boyd, Antoine Danchin, Vincent Hervé

**Affiliations:** ^1^ John Wiley & Sons Ltd Oxford UK; ^2^ Department of Microbiology and Cell Biology Montana State University Bozeman Montana USA; ^3^ School of Biomedical Sciences, li Kashing Faculty of Medicine, the University of Hong Kong SAR Hong Kong China; ^4^ Université Paris‐Saclay, INRAE, AgroParisTech, UMR SayFood Palaiseau France

*Environmental Microbiology Reports* (EMIR), along with its sister journal *Environmental Microbiology* (EMI), seek to publish research that advances knowledge on microorganisms, their function, and their interactions with the environment. The key differentiator from its sister journal, however, is that EMIR does not aim to let the subjective opinions of either editors or reviewers influence a decision on whether to publish based on the potential impact or novelty of that work. Instead, EMIR forms assessments purely based on the rigour of the methodology and the robustness of the results—an assessment of the importance of any conclusions or inferences is left to the readers.

This approach lets EMIR take a flexible view on what ‘should’ or ‘should not’ be published and aims to work with authors to ensure valuable work is as widely read as possible. Crucially, this means EMIR can address gaps in what is published and disseminated without making a call on whether it will impact journal metrics. Impact comes in many forms. Editors at EMIR look at what authors want to publish and position the journal as a venue to help them get that work noticed and widely disseminated.

With that in mind, we are launching a new article type in EMIR—***Microbiome Notes***. At both EMI and EMIR, there has been a significant increase in the number of submissions received that focus solely on microbiomes (which here we define as an examination of the microbial community present in a precisely defined environment). These papers are not necessarily hypothesis‐driven but rather are descriptive and seek to enhance the body of literature around a particular taxon or environment type. Such work is consistent with a longstanding philosophy at EMIR to publish ‘short and direct science’ (Ramos 2009). We recognise that such descriptive papers can have tremendous value to the field; building a library of microbiome work can inform and enhance more far‐reaching research by providing significantly greater context to organismal and community biodiversity.

Authors of descriptive microbiome work that is submitted to EMI will be encouraged to transfer their work to EMIR, consistent with the differing but complementary approaches taken at the two journals (refer author guidelines at both titles), and assessed purely on the rigour of the methodology and the robustness of the results. While not required, authors are encouraged to include as much contextual data associated with the studied microbiome, and these data need to be well‐curated, machine readable and following FAIR (findable, accessible, interoperable and reusable) principles (Hertz and McNeill 2024). If the editors believe that the microbial and, where appropriate, contextual data reported are sound and are an advancement in the field, the journal will consider it.

Additionally, recognising that descriptive work is often hard to align with rigid article formatting requirements, the journal will not take a position on article structure. Authors should feel free to present their microbiome studies in the best way they see fit. However, they must be consistent with journal data archiving and availability standards, i.e., data, metadata and code must be supplied at submission, and should comply with minimum standards (Jenkins et al. 2023). The International Nucleotide Sequence Database Collaboration (INSDC: DDBJ/Genbank/ENA‐EBI) is our recommended repository for sequence data, but ultimately it is up to the author as long as the chosen archive is transparent in the way it is financially supported, in the way it handles data, with a clear data structure, open and issues a permanent identifier (DOI).

## Author Contributions

**Gareth Jenkins:** conceptualization, writing – review and editing, writing – original draft. **Eric S. Boyd:** writing – original draft, writing – review and editing. **Antoine Danchin:** writing – original draft, writing – review and editing. **Vincent Hervé:** writing – original draft, writing – review and editing.

## Conflicts of Interest

The authors declare no conflicts of interest.

## Data Availability

Data sharing is not applicable to this article as no new data were created or analyzed in this study.

## References

[emi470085-bib-0001] Hertz, M. I. , and A. S. McNeill . 2024. “Eleven Quick Tips for Properly Handling Tabular Data.” PLoS Computational Biology 20, no. 11: e1012604. 39602455 10.1371/journal.pcbi.1012604 PMC11602091

[emi470085-bib-0002] Jenkins, G. B. , A. P. Beckerman , C. Bellard , et al. 2023. “Reproducibility in Ecology and Evolution: Minimum Standards for Data and Code.” Ecology and Evolution 13, no. 5: e9961. 10.1002/ece3.9961. 37181203 PMC10170304

[emi470085-bib-0003] Ramos, J. L. 2009. “Short and Direct Science.” Environmental Microbiology Reports 1: 217–219. 10.1111/j.1758-2229.2009.00048.x. 23765849

